# Deciphering the Role of Rhodanine Flanked Non-Fullerene Acceptor Molecules for Efficient Organic Photovoltaics

**DOI:** 10.3390/ijms26073314

**Published:** 2025-04-02

**Authors:** Zobia Irshad, Muzammil Hussain, Riaz Hussain, Muhammad Adnan

**Affiliations:** 1 Graduate School of Energy Science and Technology, Chungnam National University, Daejeon 34134, Republic of Korea; 2 Department of Chemistry, University of Okara, Okara 56300, Pakistan

**Keywords:** non-fullerene acceptor, rhodanine flanked, optoelectronics, organic photovoltaics

## Abstract

In recent years, extensive research has been conducted with the aim of developing non-fullerene acceptors as they have a promising ability to drive the development of cost-effective and highly efficient organic solar cells (OSCs). By harnessing the potential of rhodanine-flanked non-fullerene acceptors (NFAs), we proposed eight new A-D-A type NFAs (SBA1–SBA8) through precise end-cap modifications on both sides of the bridging-core unit. We performed various advanced quantum chemical analyses to unveil these designed materials’ potential and compared them with the synthetic reference molecule (R). The proposed NFAs series presented lower binding and excitation energy, along with narrower energy gaps of 2.11 eV and enhanced absorption at 671.20 nm and 719.88 nm in gaseous and chloroform environments, respectively. Furthermore, the optoelectronic and photophysical characterizations related to the electrostatic potential, density of states, reorganization energy of electron and hole mobilities, and transition density matrix analysis reveal that these materials could be efficiently used as acceptor materials for efficient organic photovoltaics. Additionally, to check the impact of charge transfer at the donor: acceptor (D: A) interface, we studied the PTB7-Th:SBA1 D:A analysis and demonstrated a remarkable interface charge transfer phenomenon. Therefore, the engineered SBA1–SBA8 NFAs represent a significant advancement as sustainable and effective options for developing high-performance OSCs.

## 1. Introduction

Solar photovoltaics are a promising option for meeting the world’s expanding energy needs in an environmentally beneficial way. Solar cells made from crystalline silicon are currently the industry standard for converting sunlight into electricity in commercial applications. Organic solar cells (OSCs) are thin, flexible, and cheap to produce, and have great potential as a replacement for more expensive silicon (Si) solar cells in portable power sources and integrated photovoltaics [1,2]. OSCs utilize a blended film consisting of a donor p-type organic semiconductor and an acceptor n-type organic semiconductor for their active layer. Due to the low dielectric constant of organic materials, excitons hole-electron pairs bound by strong Coulombic attraction are formed when light passes through the transparent electrode onto the active layer of an OSC [3,4,5]. Once at the donor/acceptor contacts, the excitons disperse. A recent breakthrough in solar cell technology involves the incorporation of non-fullerene acceptors as an alternative to traditional fullerene derivatives. Non-fullerene acceptors have several benefits, such as adjustable energy levels, increased electron mobility, and better absorption spectra, which make them attractive options for increasing solar cells’ total efficiency. The driving force for the switch from fullerene-based acceptors is to overcome the drawbacks of fullerene derivatives’ lower absorption coefficients, limited energy level alignment, and difficulties with synthesis [6,7,8].

We have to move towards sustainable alternatives due to the world’s increasing demand for energy. Among these, organic photovoltaics are leaders because they use organic semiconductors to harness the enormous potential of sunshine. With their carefully constructed A-D-A architecture, these molecules exhibit a fascinating synergy between alternating electron-rich donor and electron-deficient acceptor units. To aid in effective charge separation and the subsequent energy generation, think of them as little power plants. Appropriate structural alterations significantly impact the electrical characteristics of each D and A unit, which function as a molecular canvas. By strategically modulating conjugation routes, we can maximize solar energy harvesting by expanding light absorption into the hitherto unexplored near-infrared range.

Furthermore, there is exceptional control over energy levels due to the exact placement of the groups that donate and remove electrons. This results in optimal alignments of the border orbits between the molecules of the donor and acceptor, facilitating free electron transfer and improving power conversion efficiency. Non-fullerene A-D-A acceptors have an attraction beyond their remarkable ability to collect light. Their excellent solution processability opens the door to low-cost fabrication using straightforward printing methods, facilitating the development of scalable and economical OSCs. Moreover, they exhibit more excellent thermal and chemical stability than fullerenes, guaranteeing remarkable lifetime and practical endurance [9,10]. They are considered relevant for further investigation of OSC due to the non-fullerene A-D-A acceptor features, which can be valuable in terms of the search for more efficient materials for solar cells. They are designed molecules, and the science behind them is to be the building blocks in a future where roofs with solar panels transform homes into efficient little power stations. The ability of these molecules to efficiently harvest sunlight is becoming increasingly important as global demand for clean energy sources continues to grow. However, significant challenges remain in optimizing their performance, enhancing their stability, and understanding the underlying mechanisms that govern their efficiency in organic photovoltaic devices [11].

**Figure 1:**
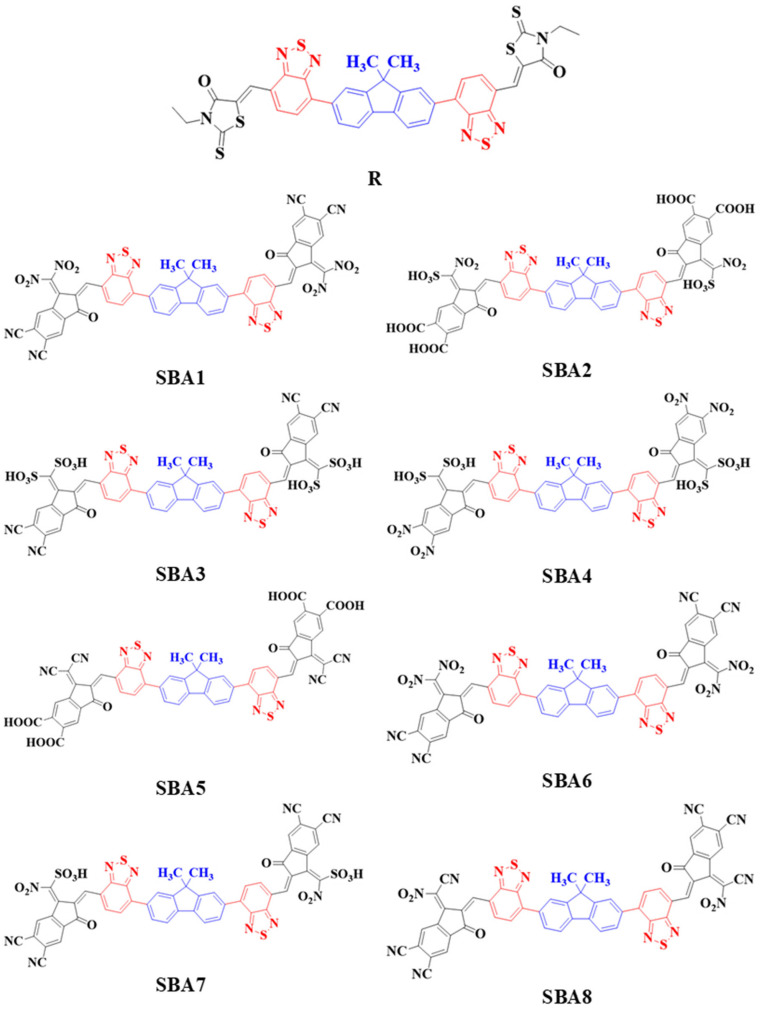
Chemical structures of newly designed SBA1–SBA8 and synthetic R molecule.

In this study, side-chain engineering of a recently synthesized rhodanine flanked-based molecule (R) is used to enhance the photovoltaic properties of modified molecules (SBA1–SBA8) [12]. The photovoltaic and optoelectronic characteristics can be altered by adjusting the end-capped moieties. These end-capped designs are shown in Figure 1. Our calculations encompassed a range of topics including binding energy (E_b_), absorption maxima (λ_max_), frontier molecular orbital (FMO), electron density difference (EDD), reorganization energy of the electrons (λ_e_), and holes (λ_h_), charge transfer analysis, the density of states (DOS), and transition density matrix analysis (TDM). We also investigated optical, geometric, optoelectronic, structural, and photovoltaic properties. To estimate the charge transfer process of the NFAs, the donor:acceptor complex study has also been investigated. Consequently, these NFAs could be effectively employed in the organic photovoltaic devices of the future.

## 2. Results and Discussion

### 2.1. Molecular Design and Chemistry of Molecules

In this study, we introduce a novel class of rhodanine flanked-based NFAs designed with superior photoelectronic properties for OSCs. The objective of this work was to theoretically develop novel rhodanine flanked-based acceptor molecules, considering potential acceptor moieties and their expected optical properties. This work used an experimentally synthesized rhodanine flanked-based FBR as the reference R molecule [12].

The reference structure, comprising a Donor and terminal acceptor, is proposed for the new study. To streamline computational efforts, we simplify the designed molecules (SBA1–SBA8 by replacing the terminal end groups with various acceptors, as shown in Figure 1. Prior studies ensured that these simplifications maintained the integrity of the molecules’ results, properties, and characteristics [13].

**Figure 2:**
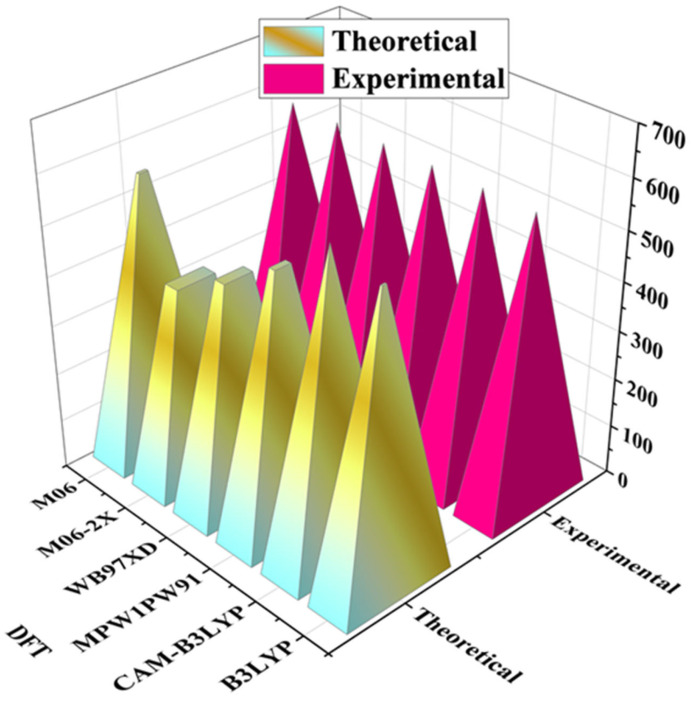
Basis set selection by applying six different DFT functionals onto the synthetic R molecule.

The λ_max_ values of the reference molecule (R) are calculated across various DFT functionals (B3LYP, CAM-B3LYP, M062X, MPW1PW91, and ωB97XD) at the 6–31G (d, p) basis set in chloroform as the solvent, using the CPCM model of different optimized molecules. The computed λ_max_ values for R are 588 nm, 450 nm, 438 nm, 634 nm, and 400 nm, respectively. Notably, the experimental λ_max_ value for R is 488 nm [14]. Figure 2 presents a 3D chart comparing experimental and DFT-computed λ_max_ values, revealing that the B3LYP/6–31G (d, p) functional exhibits excellent agreement with the experimental data and is chosen for subsequent optoelectronic calculations for both R and the designed compounds (SBA1–SBA8).

**Figure 3:**
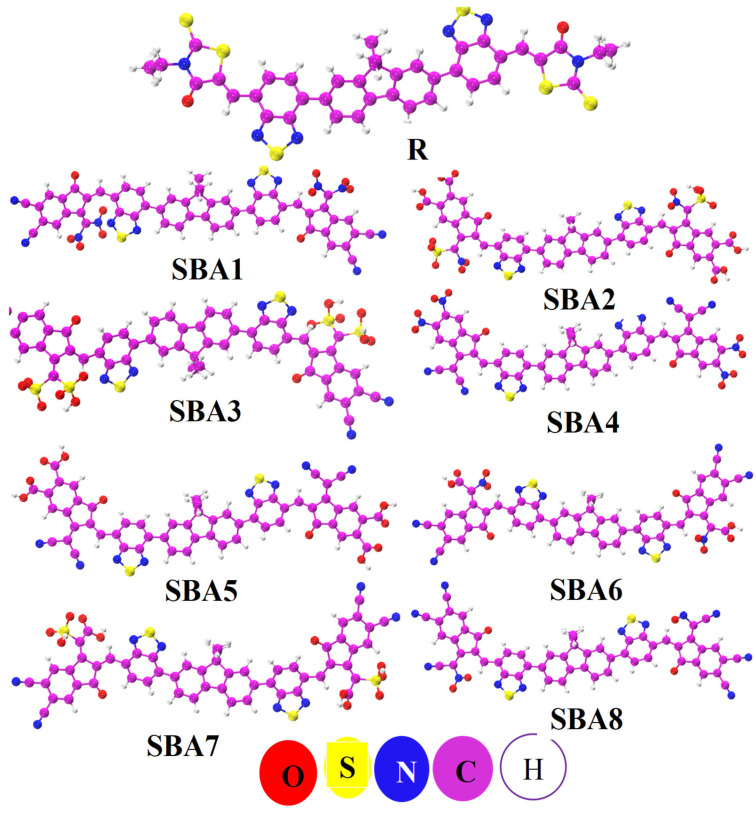
Optimized structures of designed SBA1–SBA8 and synthetic R molecule.

Utilizing the B3LYP/6–31G (d, p) function, Figure 3 showcases the optimized molecular structures of both the reference (R) and designed (SBA1–SBA8) compounds. Through the investigations of their electronic behavior, the charge transfer mechanisms and overall efficiency of different end-capped functional acceptors used, as illustrated in Figure S1, are critical for advancing OSC technologies and developing high-performance optoelectronic material.

### 2.2. Frontier Molecular Orbital Analysis

In this groundbreaking study, we meticulously examine the potential of NFAs, specifically focusing on eight distinct structures denoted as SBA1–SBA8. Renowned for their exceptional photoelectronic properties, these compounds stand as contenders for OSCs, and we assess their performance compared to the R molecule. Leveraging the computational capabilities of the B3LYP method at the 6–31G (d, p) level of DFT, we conduct a comprehensive analysis of the Highest Occupied Molecular Orbital (HOMO) and Lowest Unoccupied Molecular Orbital (LUMO) energies for each structure. Among the NFAs, SBA1 emerges as a standout performer, revealing a strategically aligned HOMO-LUMO configuration indicative of heightened charge transfer efficiency and overall stability [15]. This finding positions SBA1–SBA8 as pivotal to the efficiency landscape of organic solar cells. Band gap analysis further substantiates the superior efficiency of SBA1, showcasing the lowest band gap at 2.11 eV. This distinctive characteristic reinforces its potential to outperform its counterparts, SBA1–SBA8, and the R molecule.

In the visually compelling Figure 3, we present the optimized molecular structures of SBA1–SBA8, derived from the B3LYP/6–31G (d, p) functional, providing a clear representation of their spatial arrangements, which contribute to their enhanced performance. Examining the structures in increasing or decreasing order of performance, we observe a trend where SBA1 holds the top position, followed by SBA8, SBA4, SBA3, SBA6, SBA7, SBA2, and SBA5. This order signifies the efficiency hierarchy among the studied molecules, with SBA1 at the forefront, proving to be the most promising candidate for high-performance organic solar cells. In summary, our findings not only shed light on the individual excellence of rhodanine flanked-based NFAs but also emphasize the remarkable potential of SBA1. This study marks a significant leap towards a future characterized by more efficient and sustainable solar energy solutions.

**Figure 4:**
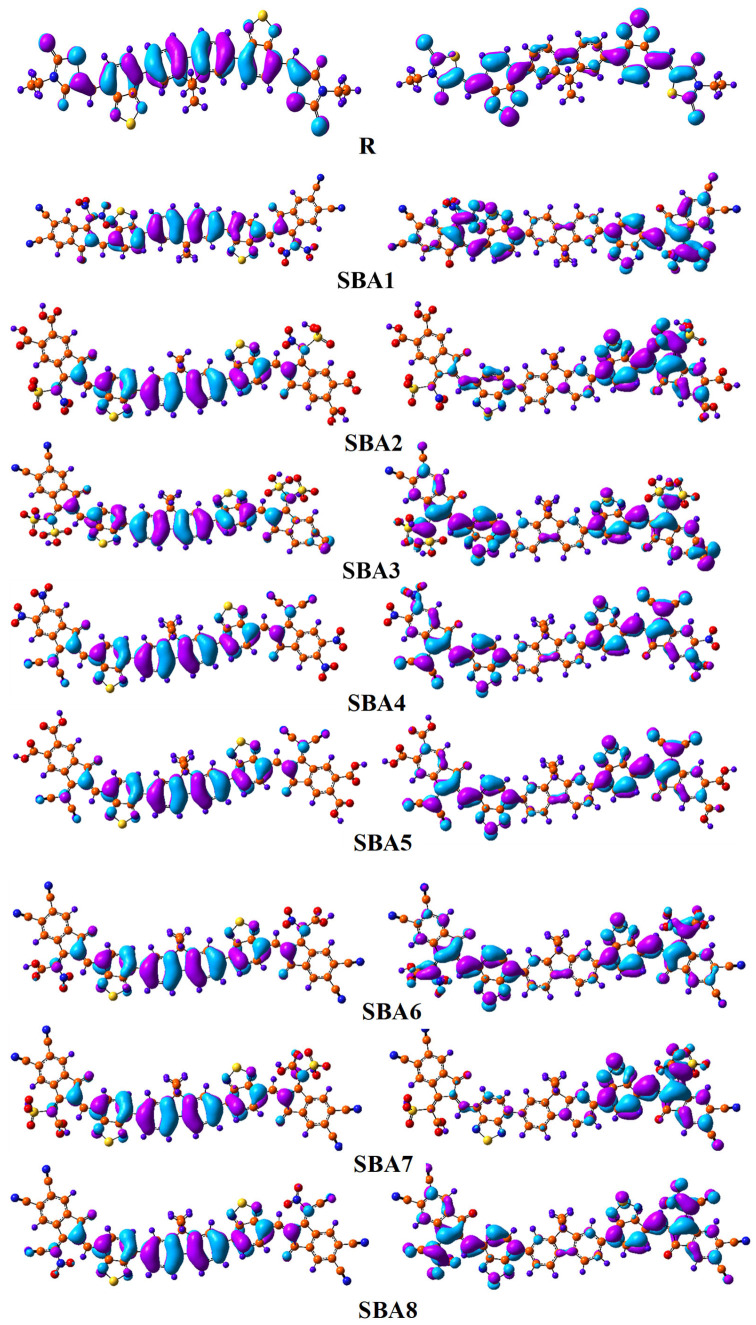
Frontier molecular orbital analysis of SBA1–SBA8 and synthetic R molecule.

The distribution pattern of HOMO and LUMO on designed molecules is shown in Figure 4. The reference molecule has HOMO and LUMO energies at −5.73 and −3.24 eV, respectively. The designed molecules SBA1–SBA8 have HOMO energy values are −6.38, −6.02, −6.22, −6.36, −6.06, −6.16, −6.26, and −6.39 eV, respectively, while LUMO energy values are observed as −4.26, −3.68, −4.03, −4.18, −3.76, −3.87, −4.01, and −4.26 eV for SBA1–SBA8, respectively. From values, it is observed that all designed molecules expressed fine values of both orbitals as compared to the R molecule. Thus, the newly formed HOMO in designed molecules is found between old LUMO and old HOMO, which efficiently narrows the HOMO–LUMO energy gap in designed molecules. The HOMO–LUMO energy gap is directly related to the transfer of charge between two molecular orbitals, i.e., a narrow band gap allows high charge transfer and vice versa. The small values of the band gap indicate that the resulting molecule has high charge transfer ability, high efficiency, and great stability. The R molecule exhibited a band gap of 2.49 eV, while designed molecules expressed band gap values as 2.11 (SBA1), 2.34 (SBA2), 2.19 (SBA3), 2.18 (SB4), 2.30 eV (SBA5), 2.29 eV (SBA6), 2.26 eV (SBA7) and 2.13 eV (SBA8). All designed molecules disclosed a narrow band gap as compared to the R molecule, which indicates that designed molecules are more efficient than the R molecule, as Table 1 shows. The decreasing order of the band gap of all studied molecules is R > SBA2 > SBA5 > SBA6 > SBA7 > SBA3 > SBA4 > SBA8 > SBA1. Based on the above order and the preceding discussion, it is confirmed that all designed molecules outperform the R molecule band gap, with SBA1 emerging as the most efficient candidate for high-performance organic solar cells. This is further supported by the 3D cone graph presented in Figure S2.

### 2.3. Density of States Analysis

**Figure 5:**
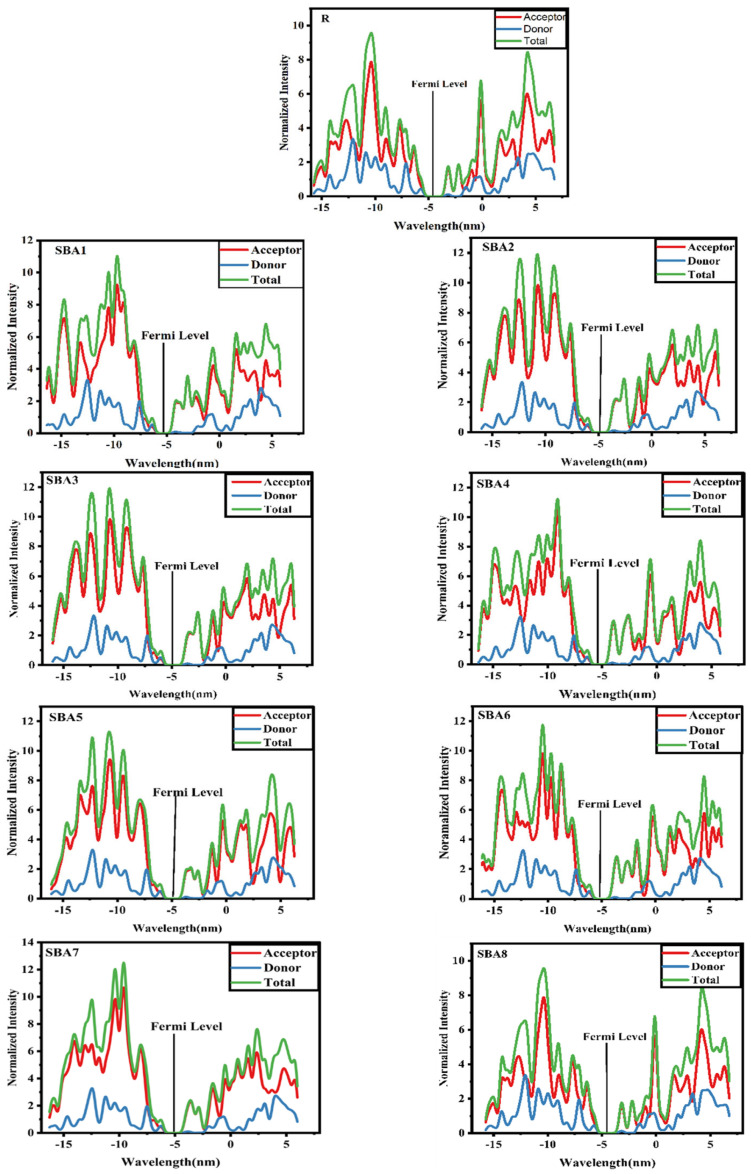
Partial density of states analysis SBA1–SBA8 and synthetic R molecule.

The density of states (DOS) plots are pivotal in assessing molecules’ electronic structure and charge transfer characteristics for organic solar cells. In our study, we employed the B3LYP method at the 6–31G (d, p) level of DFT to generate DOS plots for all studied molecules, as depicted in Figure 5. Analyzing the HOMO density shows that, for both designed and reference molecules, the major presence is on both donor units, with a lesser amount in the core unit. This distribution highlights the role of the core unit in facilitating the transfer of charge density between donor units [16,17,18,19]. Conversely, in all examined compounds, the LUMO density is found mostly on the end-capped acceptor units and, to a lesser extent, on the donor and core units. Given that Table 2 shows the percentage of charge involved in the donor and acceptor parts, this localization highlights the importance of our end-capped acceptor modifications in improving the accepting properties of these molecules, an essential component for attaining high performance in OSCs.

An understanding of the electrical structure of the molecules may be gained from the DOS plots, which give a visual depiction of the HOMO and LUMO energy levels and electron concentrations. The segment of the studied molecules (SBA1–SBA8) on the DOS graph, which consists of the donor and acceptor, is characterized by cyan-blue and red. The relative contributions of several elements to the enhancement of FMOs are shown in these lines. The full DOS is represented by a green line. The HOMO-LUMO density distribution patterns are changed in the proposed (SBA1–SBA8) molecules by the inclusion of acceptor units. A comprehensive summary of all the energy levels is provided by DOS spectra. An increase in the concentration of electronic states at the energy level is indicated by a high DOS value.

This implies that the electrons at this energy state are accessible to a range of potential energy levels. We calculated the fermi levels of each component in our investigation by summing the energies of respective FMOs. Fermi levels are used to determine whether an electron is available in the valence or conduction band. The charge may travel more efficiently when the fermi energy is close to the conducting band because electrons can jump from the valence band to the conducting band. The DOS analysis is shown graphically as an energy function. The energy level at which there is a 50% chance of finding an electron at absolute zero is known as the Fermi level (E_F_), and it is an important point of reference. Fermi level energy in the context of computed DOS plots is often determined by examining a material’s electronic structure, particularly the DOS and its relationship to the Fermi–Dirac distribution. The middle point between the highest energy level in the valence band (E_v_)-HOMO and the lowest energy level in the conduction band (E_c_)-LUMO is often where the E_F_ is found. The electron volt (eV) is the unit of measurement used to define the Fermi level. Among optoelectronic devices, the SBA1 molecule performs better photovoltaically as an acceptor molecule. Its lower valence band (i.e., HOMO) may account for its lower Fermi level. The aforementioned discussion indicates that when an alternate acceptor unit is coupled to the R, donor units in all newly proposed compounds (SBA1–SBA8) become excellent candidates for exceptionally effective OSC devices. The donor unit’s ability to donate electrons to the acceptor component is significantly increased by this attachment. This knowledge is essential for comprehending the molecules’ capacity to function as effective acceptors in organic solar cells. To summarize, the DOS plots highlight the unique electrical properties of the molecules under investigation, the thoughtful arrangement of electron densities, and our engineered molecules’ effectiveness for efficient OSCs.

### 2.4. Dihedral Angle Measurements

**Figure 6:**
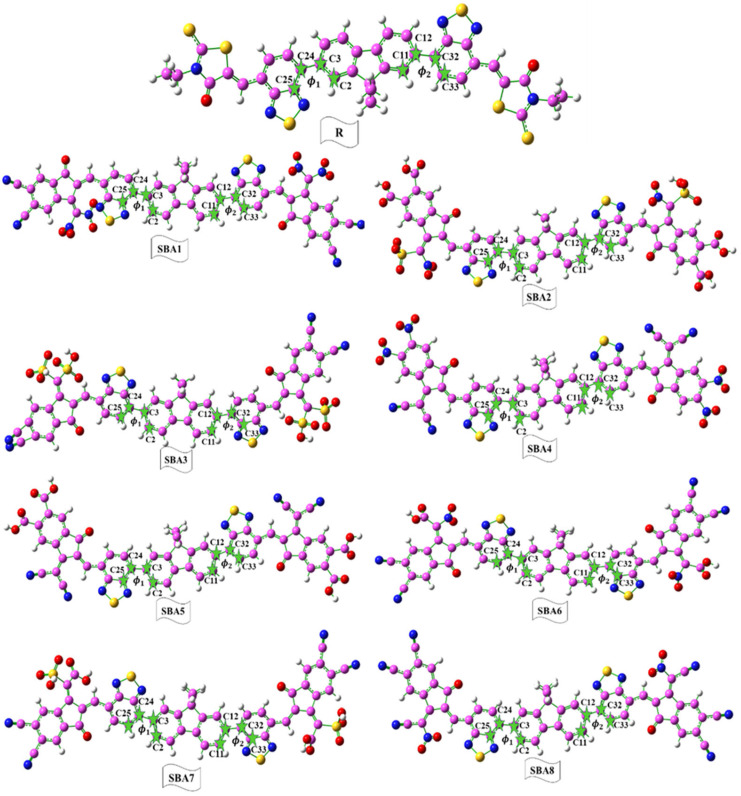
Dihedral angle of SBA1–SBA8 and synthetic R molecule.

Calculating dihedral angles is a valuable tool for estimating the optimized geometry of structure and stability after optimization, helping researchers and scientists understand the molecular structure, conformational preferences, and behavior of molecules. We calculated the dihedral angles from Gauss View 5.0.8 after optimization from Gaussian 09 software, which helped us improve the reactivity of our structural design, which might be helpful in future optoelectronic device manufacturing, as shown in Figure 6. Their values are tabulated in Table 3.

### 2.5. Optical Properties

**Figure 7:**
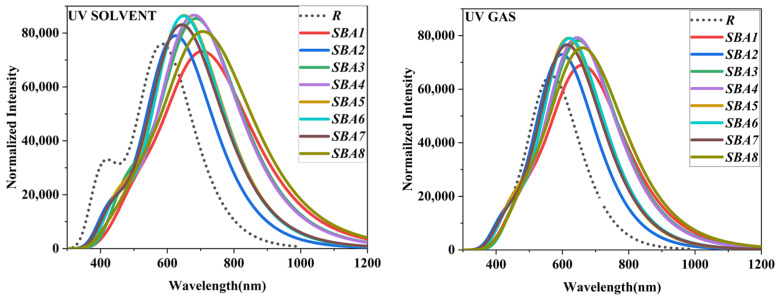
The calculated UV absorption characteristics of SBA1–SBA8 and synthetic R molecule in the solvent (O-xylene) and the gas phase.

Spectral investigations were performed on R and developed materials (SBA1–SBA8) to assess the UV/Vis absorption properties. As demonstrated in Table 4 and Table S1, UV analysis also assisted in determining the energy of excitation (E_x_). Theoretically, the λ_max_ value of R at B3LYP/6–31G (d, p) is 588.30 nm in the solvent and 569.47 nm in the gas phase. The absorption values of designed molecules in gas and solvent phases are 671.20 nm, 719.88 nm for SBA1, 611.72 nm, 643.14 nm for SBA2, 655.13 nm, 693.93 nm for SBA3, 653.93 nm, 694.12 nm for SBA4, 621.69 nm, 654.75 nm for SBA5, 625.01 nm, 653.44 nm for SBA6, 628.88 nm, 663.69 nm for SBA7 and 673.39 nm, 722.43 nm for SBA8. All of the developed molecules had visible absorption levels. SBA8 has the greatest λ_max_ in the solvent phase of all the developed compounds, followed by SBA1, SBA4, SBA3, SBA6, and SBA2 [20]. The end-capped group demonstrated a greater electron withdrawing action than R. Because of the presence of nitro groups, sulphonic groups, carboxylic groups, and cyan groups, SBA1, SBA4, and SBA8 displayed the greatest electron withdrawing impact of all the developed compounds [21,22]. All of our developed compounds had a larger redshift value. Additionally, the enhanced absorption in the visible spectrum improves the quantity of photo-light absorbed by the photoactive layer and is thought to improve OSCs’ photovoltaic capabilities. Sunlight absorption occurs in the active part of a bilayer organic photovoltaic (OPV) cell, which is made up of donor and acceptor materials that are semiconducting organic compounds. This process is known as photocurrent production. The donor material (D) is in charge of providing electrons and initiating the primary transit of holes, whereas the acceptor material (A) is in charge of electron retraction and primary transport. The molecules showed a maximum absorbance (λ_max_) that was within the wavelength range of 643.14–722.43 nm in a solvent. Assigning the π-π* transition of the molecules to these peaks, which exhibited considerable energy. This transition occurs as a result of unsaturation present on both sides of the molecules. Figure 7 depicts the absorption spectra of R and newly proposed compounds (SBA1–SBA8) in the solvent and gas phase.

The solvent phase may be more beneficial during blend formation since oscillator strength (*f*_os_) values therein higher than those in the gas phase. This is due to the possibility that the solvent phase will improve photon absorption, which will improve the materials’ optical characteristics. Alternatively, we may say that the material absorption phenomenon and the *f*_os_ values are directly correlated. Therefore, higher values of *f*_os_ are ideal for organic semiconducting materials. As a result, all of the materials we have created in this context have good and comparable values for the solvent and gas phase in both media, proving the effectiveness of our design strategy for building a more durable photovoltaic system. Lower excitation energy (E_x_) values are typically linked to higher optical conductivity (OSC) abilities. The electron density allocation from HOMO to LUMO is also made easier by low E_x_. E_x_ is calculated theoretically for every molecule in the solvent and gas phases, as mentioned in Table 4 and Table S1.

All the molecules that have been found have low excitation energy and strong oscillations. The indicated molecules (SBA1–SBA8) have excitation energies of 1.72, 1.93, 1.79, 1.79, 1.89, 1.90, 1.87, and 1.72 eV in the solvent phase, and 1.85, 2.03, 1.89, 1.90, 1.99, 1.98, 1.97, and 1.84 eV in the gas phase. All of the proposed compounds have comparable excitation energies, indicating that they could be viable candidates for the more effective OSCs.

### 2.6. Transition Density Matrix Analysis

**Figure 8:**
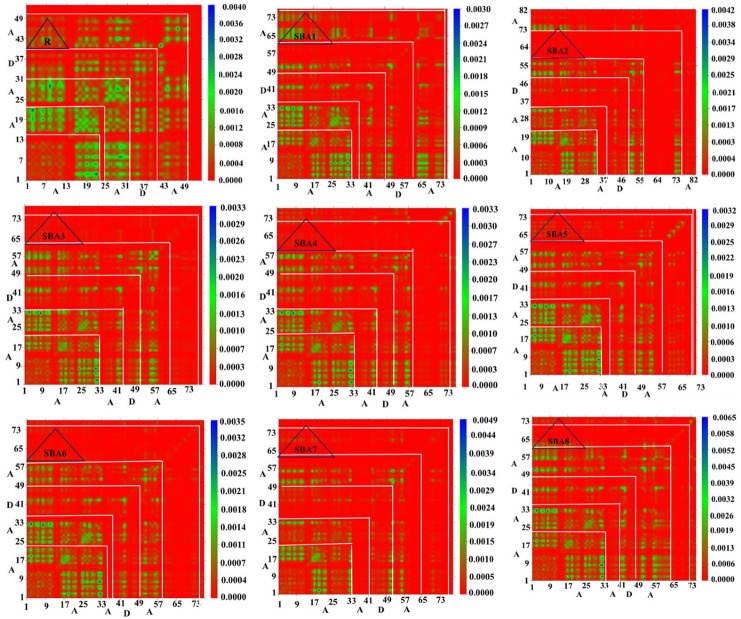
Transition density matrix plots of SBA1–SBA8 and synthetic R molecule.

The transition types in the R molecule and the synthesized compounds (SBA1–SBA8) were analyzed using TDM matrices. Figure 8 presents the results of a calculation utilizing the B3LYP and 6–31G (d, p) basis set, yielding TDMs for the emission and absorption of all suggested compounds in the atmosphere. The contribution of hydrogen atoms has been neglected due to their small effect on transitions. Excitation of electrons, hole-electron localization, and interaction in the excited state between acceptor and donor groups can only be estimated using the TDMs method, which is why it is so crucial. In addition, these reveal the existence of multiple charge transfers between the excited states. Electronic transitions have an impact on the creation and migration of excitons, which are pairs of electrons and holes, throughout the materials research. A common explanation for the movement of excitons toward the donor–acceptor contact is material efficiency. Because coherent excitons are spatially delocalized, they can travel farther without losing their excitation energy. This delocalization is crucial because it governs the effective diffusion of excitons in the solar cell’s active layer. We divided our R and proposed molecules (SBA1–SBA8) into Acceptor and Donor segments so that we could examine these patterns.

According to the TDM diagrams, molecules R, SBA2, SBA3, SBA6, and SBA8 demonstrate electron coherence primarily within the donor and along the diagonal of the acceptor region. In contrast, for SBA1, SBA4, SBA5, and SBA7, electron coherence is predominantly located in the acceptor region, with a smaller extent observed in the donor region. The TDM plots for R and the newly designed molecules (SBA1–SBA8) indicate efficient electron transfer from the donor to the acceptor without electron trapping. Critical energy analysis reveals that the proposed molecule SBA1 has the highest concentration of easily dissociable charges. This finding suggests that, among the studied molecules (R and SBA1–SBA8), SBA3 exhibits the most efficient charge distribution. Moreover, the designed compounds (SBA1–SBA8) display a greater capacity for charge distribution than R, indicating a significant potential to enhance the current charge density (*J*_sc_). Among the tested molecules, SBA3 is identified as the most effective in charge dispersion, which is likely to have a substantial impact on increasing *J*_sc_.

### 2.7. Binding Energy Measurement

Binding energy (E_b_) can be quantified commonly as the energy needed to pull apart an electron-hole pair or exciton into free charges, electrons, and holes. This energy is highly valuable for assessing the degree of charge separation in organic solar cells particularly when aligned with NFAs. It helps assess the optoelectronic properties, exciton dissociation energy, and overall efficiency of OSCs. The E_b_ can be used to quantify the coulombic attraction between an electron and a hole. There is a direct relationship between the coulombic force in an electron-hole pair and the E_b_; as the exciton dissociation in the excited state increases, both the coulombic interaction and the E_b_ decrease. A higher E_b_ value indicates reduced exciton dissociation in the excited state and a stronger coulombic interaction between the electron and the hole. The binding energy (E_b_) of R and developed compounds (SBA1–SBA8) are calculated using the following Equation (1). The formula for E_b_ is:

(1) $$E_{b}=E_{H-L}-E_{opt}$$

The E_H-L_ in Equation (1) represents the energy gap between the HOMO and LUMO states, and the E_opt_ represents the minimal energy needed for initial excitation from the ground state (S_0_) to the first singlet excited state (S_1_) through the formation of an electron and hole pair. The calculated E_b_ values for the reference molecule R and the newly developed compounds SBA1–SBA8 are presented in Table 5. The reference molecule R exhibits an E_b_ value of 0.31 eV. Interestingly, the E_b_ values for the newly proposed SBA1–SBA8 compounds are lower and closely aligned with that of R.

### 2.8. Molecular Electrostatic Potential Analysis

**Figure 9:**
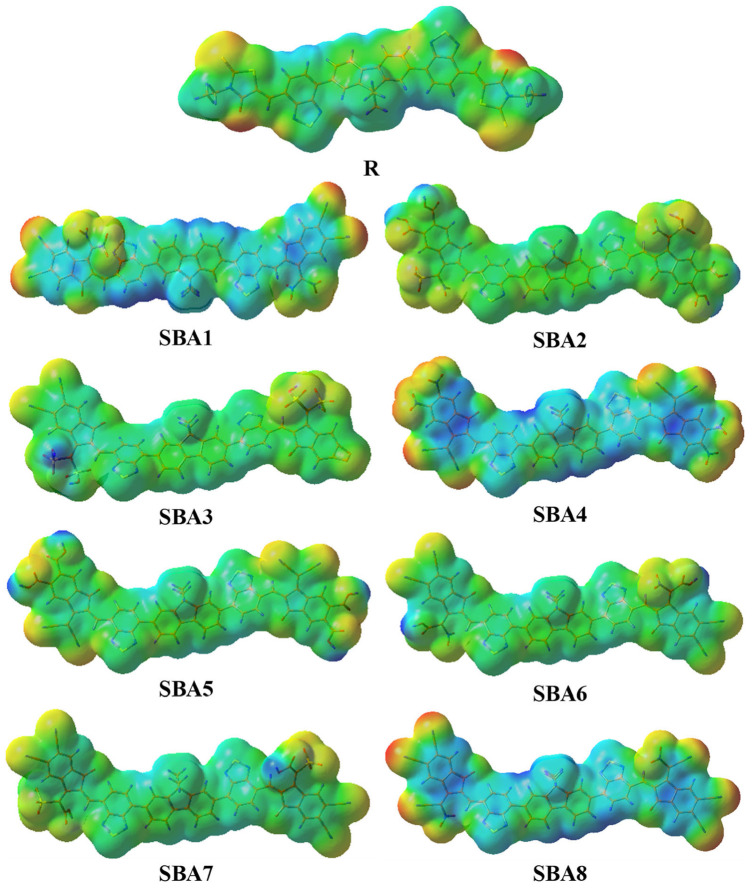
Molecular electrostatic potential plot of SBA1–SBA8 and synthetic R molecule.

Anticipating the existence of dynamic charging sites in the materials requires the application of molecular electrostatic potential (MEP) analysis, a technique that determines the various charge positions in materials. Each distinct charging area has an important illustration related to the specific properties of the material. The materials frequently comprise different charged sites and combinations of charged places. The operational capabilities of the materials are significantly impacted by these factors. Figure 9 therefore shows the computed MEP plots for R and the suggested series (SBA1–SBA8) at B3LYP/6–31G (d, p).

The separations between different color patterns and color mixes are displayed in these MEP spectra, where each color represents a distinct property of the material. For instance, the material’s neutral zone is shown in green, its electropositive region is shown in blue, and its negative half is shown in red. Yellow colors represent Transition zones between negative and positive potentials. These areas may indicate regions of moderate electron density. The produced molecules (SBA1–SBA8) impressively display color patterns that correspond with R, proving the effectiveness of our materials modeling methodology. Moreover, the presence of discrete charged spots indicates the unique property of that material that helps determine their concealed propensity for effective OSCs. As a result, these MEP plots could be helpful and provide a better means of obtaining comprehensive data on suggested materials. As such, it exemplifies our effective materials design and characterization process to investigate the influence of adjusting different functional groups to produce molecules with improved characteristics. Perhaps, in the future, we should focus on developing these components to produce efficient OSCs. These MEP investigations further confirm previous findings and demonstrate the potential for effective OSC preparation using the SBA1–SBA8 planned asymmetric series.

### 2.9. Quantum Chemical Parameters

The kinetic stability and chemical reactivity of R and all designed molecules (SBA1–SBA8) have been calculated, and the results are displayed in Table 6.

(2) $$Chemical\;hardness\;(\eta )=\frac{\left(E_{LUMO}-E_{HOMO}\right)}{2}$$

(3) $$Chemical\;softness\left(S\right)=\frac{1}{\eta }$$

Chemical hardness and softness values are calculated using the above equations [23]. All designed molecules (SBA1–SBA8) demonstrate soft values due to lower bandgap values than R.

(4) $$Chemical\;potential\left(\mu \right)=\frac{\left(E_{HOMO+}E_{LUMO}\right)}{2}$$

The above equation calculates the chemical potential of all designed molecules [24]. It describes the electronic cloud escaping capability (8). Freshly designed molecules (SBA1–SBA8) have higher negative chemical potential values than R. Higher negative values indicate that designed molecules (SBA1–SBA8) are highly reactive and cannot decompose easily, even if they have stable compounds.

(5) $$Electronegativity\left(\chi \right)=-\frac{\left(E_{HOMO+}E_{LUMO}\right)}{2}$$

(6) $$Electronegativity\left(\chi \right)=-\frac{\left(E_{HOMO+}E_{LUMO}\right)}{2}$$

**Figure 10:**
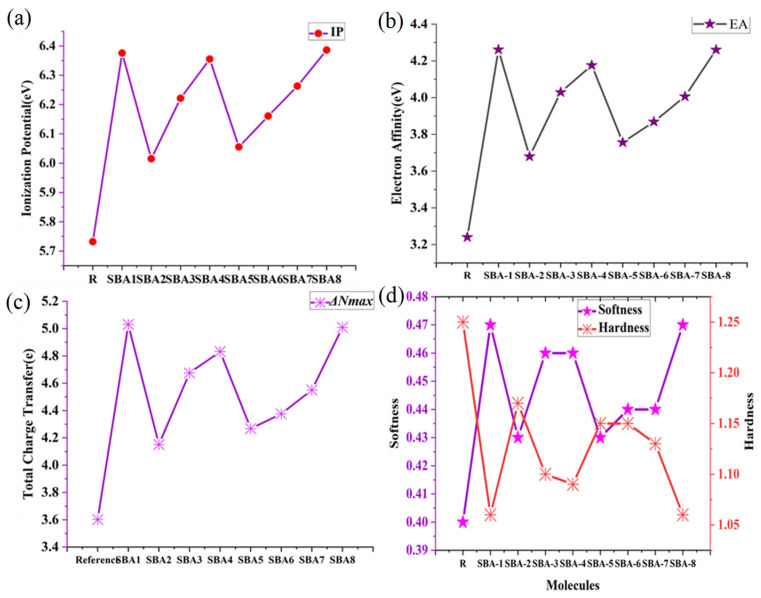
The calculated analysis of (**a**) ionization potential, (**b**) electron affinity, (**c**) total charge transfer, and (**d**) softness and hardness as a reactivity parameter of SBA1–SBA8 and synthetic R molecule.

Electronegativity and electrophilicity have been calculated for designed molecules by using Equations (5) and (6) [25]. Values listed in Table 6 displayed that all molecules (SBA1–SBA8) manifested higher values of electrophilicity index and electronegativity than R, indicating that designed molecules are accompanied by strong electron-withdrawing moieties and their chemical reactivity parameters [26] as ionization potential (a), electron affinity (b), total charge transfer (c) and softness and hardness (d) is shown in Figure 10.

(7) $$Total\;amount\;of\;charge\;transfer\left(∆Nmax\right)=-\frac{\mu }{\eta }$$

### 2.10. Reorganizational Energy Measurements

**Figure 11:**
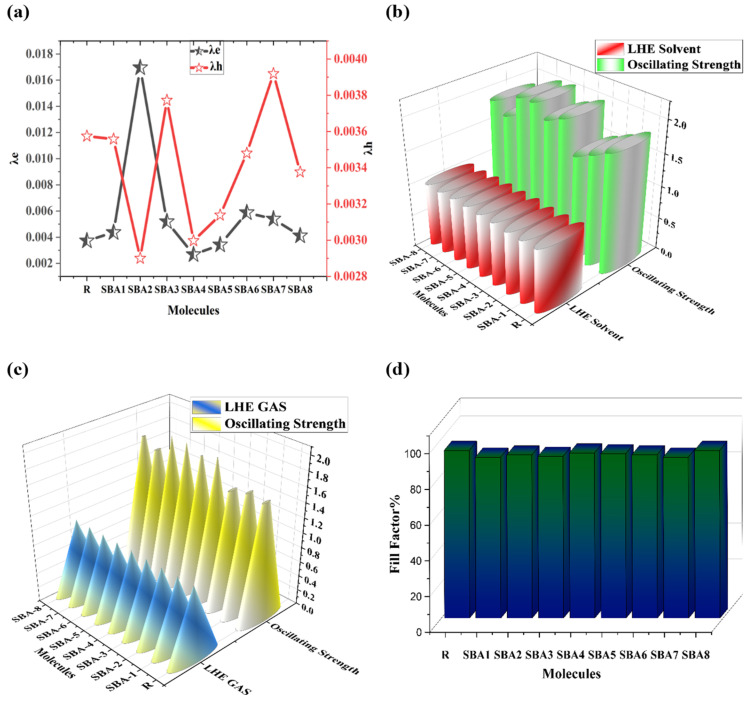
The calculated (**a**) electron (λ_e_) and hole (λ_h_) reorganization energies, (**b**) light harvesting efficiency in the solvent, (**c**) light harvesting efficiency in the gas phase, and (**d**) calculated fill factor values of SBA1–SBA8 and synthetic R molecule.

To evaluate the charge mobility efficiency conditions of solar cells (SCs), the hole and electron mobility through excitation is predominantly determined by the reorganization energy equation. The computation of charge distribution from the acceptor end-capped to the donor aids in understanding these dynamics. Materials exhibiting strong photovoltaic effects and high charge mobility tend to have lower reorganization energy values (Figure 11a). In the case of solar cells, two types of reorganizational energy have been identified: internal reorganizational energy (λ_int_) and external reorganizational energy (λ_ext_) [27].

The Marcus equation [28,29,30] is employed to assess hole mobility (λ_h_) and electron mobility (λ_e_), with a primary focus on λ_int_. Comparative analysis between R and the newly designed compounds (SBA1–SBA8) reveals reorganizational hole and electron energies, as Visualized in Figure 11a. For the R molecule, λ_e_ and λ_h_ yield values of 0.0037 and 0.0036, respectively. In terms of electron mobility (λ_e_), the decreasing order is SBA2 > SBA6 > SBA7 > SBA3 > SBA1 > SBA8 > R > SBA5 > SBA4. Notably, SBA5 and SBA4 exhibit superior electron mobility prospects with lower λ_e_ values than R. The observed order for hole mobility (λ_h_) is SBA7 > SBA3 > R > SBA1 > SBA6 > SBA8 > SBA5 > SBA4 and SBA2, indicating that SBA1, SBA6 > SBA8 > SBA4 > SBA2 have enhanced hole mobility.

The discussion of reorganizational energy reveals that the proposed series (SBA1–SBA8) possesses actual hole and electron mobility characteristics aligned with R, with substantially lower electron and hole levels than R, as presented in Figure 11a. These improvements stem from the effective end-capped engineering of the molecules, incorporating beneficial functional group entities (-NO_2_, -COOH, -F, -CN, and -SO_3_H), thereby enhancing the materials’ photovoltaic capabilities.

### 2.11. Light Harvesting Efficiency Analysis

The light-harvesting efficiency (LHE) of a photovoltaic molecule is a measurement of its capacity to absorb solar radiation and transform it into electrical energy. LHE thus quantifies the ability of the absorbed photons to be used to produce excited states in the active layer of a solar cell. The strength of oscillator *f*_os_ ultimately affects the creation of *J*_sc_ and is highly associated with LHE. Thus, a major factor influencing solar devices’ efficiency is their ability to collect sunlight. The *Jsc* value is calculated using Equation (8) and is used in the production of solar cell devices [31].

(8) $$J_{SC}=\int_{\lambda }^{0}LHE\left(\lambda \right).\phi_{inj}.\eta_{collect}.d\lambda$$

In this instance, η_collect_ stands for the overall charge collection, and ϕ_inj_ indicates the electronic injection efficiency. The *J*_sc_ plays a major role in the photovoltaic efficacy of solar cells. The maximum current density that a solar cell is capable of producing is measured in the absence of external resistance. *J*_sc_ is the maximum current that, under certain conditions, may be drawn from the solar cell. For the substances that are currently being studied, LHE was calculated using Equation (9). There are values in both the solvent and gas phase are presented in Table S2, the relation of LHE and *f*_os_ in the gas phase is shown in Table S3, and their graph is shown in solvent and in gas (Figure 11b,c).

(9) $$LHE=1-10^{-f}$$

All molecules under investigation show comparable LHE as compared to R. Modified molecules SBA3 (0.9905 eV), SBA5 (0.9917 eV), and SBA6 (0.9913 eV) exhibit the highest LHE in the solvent due to their stronger *f*_os_ compared to other compounds. All newly introduced compounds have comparable LHE values, which satisfies one of the requirements needed to create future effective devices.

### 2.12. Photovoltaic Properties

Open circuit voltage (*V*_oc_), a vital metric for evaluating organic solar cell performance, symbolizes the pinnacle of current that can be harnessed from an optical device. It is, essentially, the zero-current maximum voltage attainable from any such device. The intricacies influencing *V*_oc_ encompass external fluorescence efficiency, charge-carrier recombination, electrode work functions, light source characteristics, solar cell temperature, light intensity, environmental factors, and the properties of the materials involved. The disparity between the HOMO and LUMO energies of donor and acceptor molecules stands as a key determinant of *V*_oc_. Attaining higher *V*_oc_ entails a delicate balance: the donor molecule’s HOMO level should be sufficiently low, while the acceptor molecule’s LUMO level should be elevated. In our foray into quantum chemical exploration, we embarked on a calculation of *V*_oc_ for both (R) and the designed molecules (SBA1–SBA8), employing two distinguished acceptor molecules for comparison. Initially, we scrutinized the LUMO energy levels of R and SBA1–SBA8, aligning them with the HOMO energy level of the renowned donor polymeric material PTB7-Th. Subsequently, we turned to a recently acclaimed acceptor molecule, boasting record-breaking efficiency, to benchmark *V*_oc_ results.

**Figure 12:**
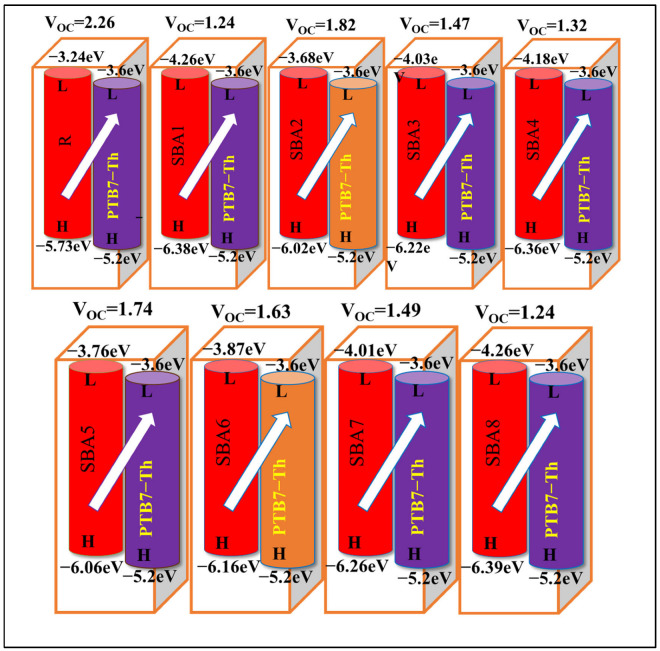
The calculated open circuit voltages of SBA1–SBA8 and synthetic R molecule with donor polymer PTB7-Th.

Theoretical values of *V*_oc_ were derived using the Scharber Equation (10), revealing comparable *V*_oc_ values among all investigated compounds [32,33]. The pecking order of open circuit voltage concerning the HOMO donor–LUMO acceptor energy gap mirrored the reverse LUMO energy level order: R > SBA2 > SBA5 > SBA6 > SBA7 > SBA3 > SBA4 > SBA1 > SBA8. Intriguingly, the *V*_oc_ values of SBA1 and SBA8 are the same (1.24 V). This suggests that these molecules have similar performance in terms of their *V*_oc_, which is a critical parameter in determining the efficiency of a solar cell. To visually articulate the energy levels and transitions critical for enhancing the power conversion efficiency of solar cells, *V*_oc_ diagrams of R and the designed molecules (SBA1–SBA8) with PTB7-Th donor molecules are presented in Figure 12, and *V*_oc_ values are tabulated in Table 7. When combined with PTB7-Th polymer donors, the proposed acceptor molecules (SBA1–SBA8) exhibit tremendous promise for use in solar cells. Strong performance is indicated by the *V*_oc_ values, which are comparable to R. They therefore present a good option for effective charge transfer. All things considered, SBA1–SBA8 are good materials for enhancing solar cell performance [34].

(10) $$V_{oc}=\left.\left|E_{HOMO}^{D}\right.\right|-\left|\left.E_{LUMO}^{A}\right|\right.-0.3$$

The power conversion efficiency (PCE) is an essential input variable that demonstrates the conversion efficiency of a solar cell facilitating the generation of electrical power from sunlight. It established the relationship between the electrical power produced to the incident solar power. PCE is calculated through Equation (11) and is shown in Figure S3 their values are mentioned in Table 7 [35] and their fill factor values are shown in Figure 11d.

(11) $$PCE=\frac{JscV_{oc}FF}{P_{in}}$$

Theoretically, PCE is frequently anticipated by calculating important parameters like V_oc_, *J*_sc_, and fill factor (FF) based on the electronic properties, optical properties (like LHE), and charge transport characteristics (like exciton binding energy and reorganization energy) of the material. The intention is to find materials with high PCE that can help in the development of solar cells with high efficiency. The PCE of our theoretically designed molecules (SBA1–SBA8) is higher than R, which indicates that our structural design is efficient for the conversion of sunlight into electrical energy.

The fill factor has a major impact on the PCE of organic photovoltaic (PV) devices. The primary aspect influencing it is the Voc value. Higher *V*_oc_ values boost the FF and considerably increase the efficiency of the system. FF can be computed using Equation (12), and values are given in Table 7.

(12) $$FF=e\frac{V_{OC}}{K_{B}T}-\frac{\ln\left(e\frac{V_{OC}}{K_{B}T}+0.72\right)}{\frac{eV_{OC}}{K_{B}T}+1}$$

When temperature is expressed as 298 K, e is the elementary charge (fixed at 1), and K_B_ is Boltzmann’s constant (8.61733034 × 10^5^). All designed molecules (SBA1–SBA8) show comparable FF values with R, as indicated in Table 7, proving an effective approach for enhancing the molecule’s photovoltaic properties.

### 2.13. Electron Density Difference Analysis

The electronic properties (SBA1–SBA8) designed molecules were evaluated by comparing key parameters with the R, as detailed in Table 8. The Hole Distribution Index (HDI) and Electron Distribution Index (EDI) values highlight significant variations in the energy levels associated with hole and electron distributions across the molecules. SBA4 exhibited the lowest HDI (3.45 eV) and EDI (4.28 eV), which indicates lower energy associated with both hole and electron distributions compared to R (3.79 eV and 4.52 eV, respectively). Lower HDI and EDI values generally suggest a more favorable distribution of charge carriers, which could lead to improved charge transport properties. Conversely, SBA1 showed the highest HDI (4.32 eV) and EDI (11.2 eV), suggesting higher energy levels and potentially more localized charge distributions, which could hinder efficient charge transport. The integral hole and integral electron values represent the total charge distribution within each molecule. SBA7 and SBA4, with lower integral hole (0.7801 amu and 0.8113 amu) and electron (0.7797 amu and 0.8116 amu) values compared to R (0.9696 amu for both), suggesting less overall charge distribution, which may imply less effective charge separation. This could reduce the efficiency of charge transport. On the other hand, SBA8 and SBA6 showed higher integral values, with SBA8 having the highest integral hole (0.9347 amu) and electron (0.9349 amu) values, indicating a more extensive charge distribution. This might enhance the charge separation and transport, leading to improved device performance.

Integral Transition Density (TD) values indicate the balance between hole and electron distributions. R displayed a slightly positive integral TD (0.00016 amu), which suggests a minor favoring of electron density. Molecules like SBA1 and SBA5, with negative TD values (−0.00015 amu and −0.00009 amu), suggest a shift towards hole dominance, potentially leading to better hole transport properties. However, SBA8, SBA4, and SBA7, with very low positive TD values (0.00002, 0.00007, and 0.00008 amu), indicate balanced or slightly electron-favored charge distributions, which might enhance the balance in charge transport.

**Figure 13:**
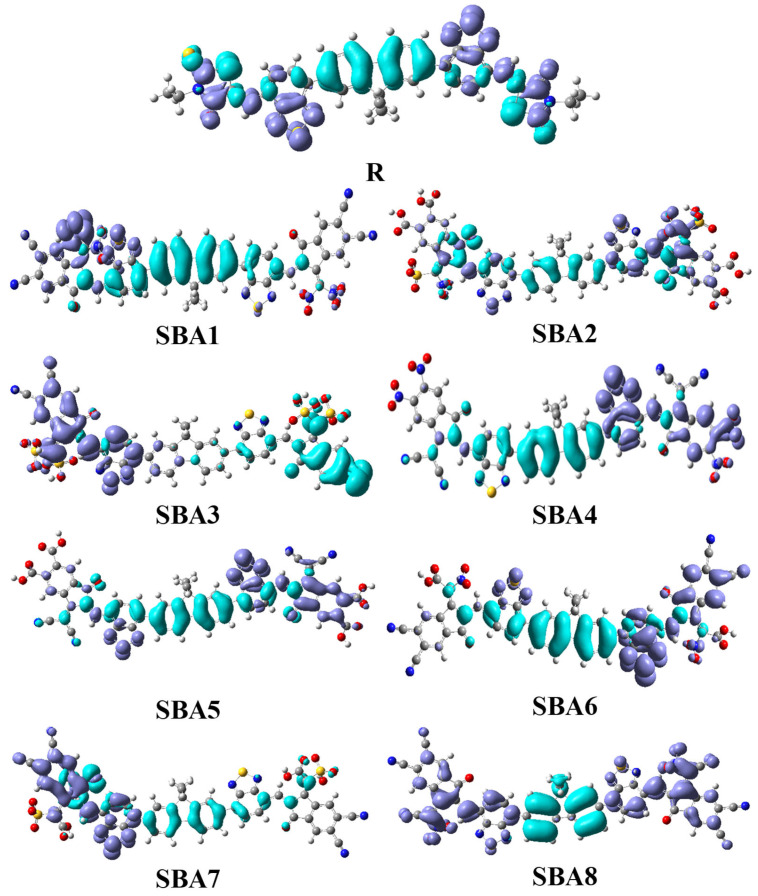
The calculated electron density difference maps of SBA1–SBA8 and synthetic R molecule.

The transfer index (t Index) reflects the energy differences associated with charge transfer within the molecules. SBA3, with a highly positive t Index (10.64 eV), indicates significant energy differences favoring charge retention, which could lead to poor charge transport efficiency. Molecules like SBA2, SBA5, and SBA7, with more negative t Index values (−5.93 eV, −4.35 eV, and −1.60 eV) compared to R (−1.84 eV), suggest greater energy differences and more effective charge transfer processes. A less negative or near-zero t Index, as seen in SBA1 (0.93 eV) and SBA6 (−0.63 eV), may imply moderate charge transfer dynamics, potentially balancing between retention and transport. The H Index represents the energy associated with hole dynamics. A higher H Index, as seen in SBA2 (9.11 eV), compared to R (8.67 eV), indicates more energy involved in hole dynamics, which might enhance hole mobility. SBA7, with the lowest H Index (6.76 eV), suggests lower energy in hole dynamics, potentially reducing hole mobility. The D Index is related to electron dynamics, with higher values indicating greater energy associated with electron movement. SBA3, with a significantly higher D Index (17.7 eV) compared to R (0.40 eV), suggests much greater energy in electron dynamics, which could enhance electron mobility but might also lead to instability. SBA4 and SBA1, with comparable D Index values (6.98 eV and 6.97 eV), suggest a balanced approach, which could support both stability and efficient electron transport. Finally, the hole charge transfer (HCT) values indicate the energy involved in hole transfer. Higher HCT values, as seen in SBA2 (8.66 eV) and SBA5 (7.70 eV), compared to R (2.24 eV), indicate more energy involved in hole transfer, which could enhance hole mobility and overall device efficiency. SBA8 and SBA3, with lower HCT values (3.29 eV and 7.08 eV), suggest less energy involved, which might reduce hole mobility but could improve stability. Therefore, the comparative analysis of these electronic properties, relative to the R, highlights variations in electron density, charge transfer characteristics, and energy dynamics that can significantly influence the performance of organic solar cells as their plots are depicted in Figure 13. These differences underscore the unique electronic properties of each molecule, which are crucial for optimizing their application in organic solar cells.

### 2.14. Natural Population Analysis

**Figure 14:**
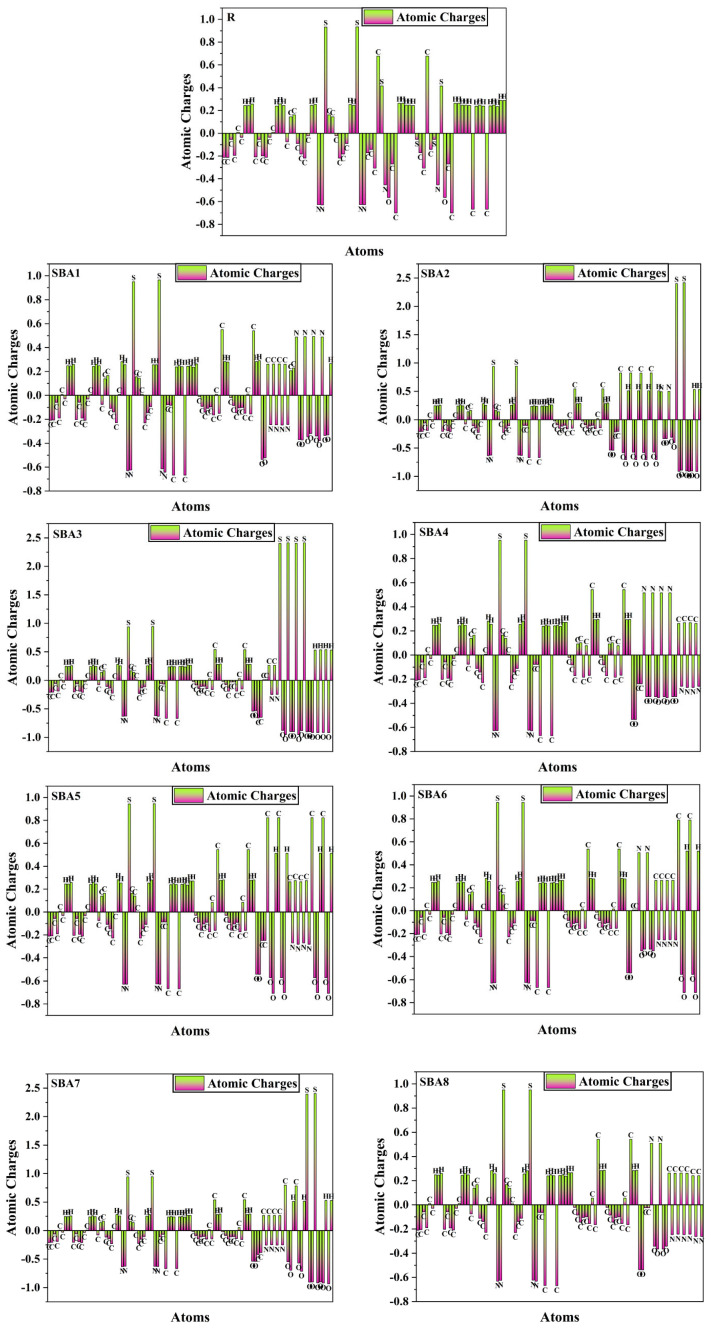
Plots of NPA for all investigated SBA1–SBA8 acceptors along with reference R.

Natural population analysis (NPA) provides information to detect atomic charges and electron distributions within molecular complexes. In Figure 14, NPA calculations reveal net atomic charges for rhodanine-flanked norfullerene acceptor molecules. The charge of hydrogen atoms becomes positively charged because they lie adjacent to sulfur, nitrogen, and carbon atoms in the structure. Most carbon atoms found in both the donor and acceptor regions possess negative charges, but components bound to fluorine, nitrogen sulfur, and oxygen atoms remain positively charged.

Whenever nitrogen connects to carbon or hydrogen atoms, they obtain a primarily negative electronic charge. The nitrogen atom in compounds SBA1, SBA4, and SBA8 carries a positive charge because it is bonded to oxygen. Like hydrogen and carbon atoms, oxygen atoms typically possess an electron deficit. Electronegative bonding between sulfur atoms and nitrogen or oxygen molecules gives sulfur a positive charge and appears in areas characterized as both donors and acceptors. The negative charge in rhodanine-flanked structural high-throughput materials extends from nitrogen, oxygen, fluorine, and carbon after considering all active centers (R and SBA1–SBA8). Electron transfer kinetics from donor to acceptor depends heavily on the asymmetric distribution of positive charges from nitrogen, sulfur, and hydrogen together with carbon-based atoms. These molecules showcase beneficial characteristics for solar cell device fabrication, and additional changes to the acceptor segment would lead to improved charge-splitting abilities.

### 2.15. Quadrupole Moment (Q_20_)

**Figure 15:**
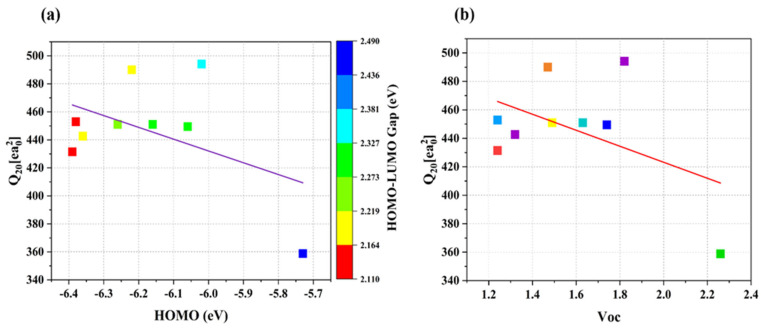
(**a**) Plot of the Q_20_–HOMO correlation for the NFA complexes under investigation. Each point’s hue corresponds to its matching HOMO–LUMO gap value, (**b**) Plot of the Q_20_–V_OC_ connection for the NFA complexes under investigation. The matching (LUMO+1)–LUMO gap value is indicated by the color of each point.

Evaluation of the database focused on the relationship between vital calculated parameters, where Q_20_ served as a critical factor for charge separation and recombination across the donor–acceptor interface while impacting short-circuit current and power conversion efficiency measures in solar cells [36]. Higher Q_20_ values create larger J_SC_ output, yet extreme Q_20_ levels make the open-circuit voltage (V_OC_) suffer which demands optimization of J_SC_ and V_OC_ together [37]. A larger HOMO–LUMO gap shows an opposite relationship with Q_20_ based on data presented in Figure 15a which produces lower values of Q_20_. Non-fullerene electron-withdrawing acceptor units in A-D-A structures lower the HOMO–LUMO gap, which allows better charge flow while producing higher molecular quadrupole moments. The alteration of end-capped in all designed NFAs (SBA1–SBA8) results in simultaneously significant HOMO/LUMO energy levels and improved quadrupole moment values which yield enhanced final device performance. The research presented in Figure 15b demonstrates that Q_20_ decreases as V_OC_ increases consistent with findings in existing materials science [38]. NFAs featuring V_OC_ ratings between 1.24 and 1.82 V demonstrate superior Q_20_ values and diminished ΔLUMO gaps which helps donors benefit from LUMO-based electron reception to boost PCE metrics [39]. Systems with small dipoles across the LUMO energy levels possess optimal charge transfer states that enhance both PCE and charge separation efficiency [40]. More than half of the NFAs maintain a ΔLUMO at or below 0.3 eV, which represents an advantageous condition for top-performing electron acceptor systems. Calculated Q_20_ values of all molecules are given in Table 9. Successful optimization of Q_20_ and ΔLUMO values emerges as crucial for effective NFA design because these values directly affect charge separation and resulting J_SC_ and V_OC_ performances in OSCs. The rhodanine-flanked functionalization achieves dual benefits by improving device stability as well as minimizing energy waste and enhancing performance. Small values of ΔLUMO establish high-energy donation states that enhance efficient charge transport within the device fabrication. Future research efforts should develop supplemental functionalization techniques as a means to enhance device efficiency by fine-tuning electronic properties.

### 2.16. Charge Transfer Analysis

**Figure 16:**
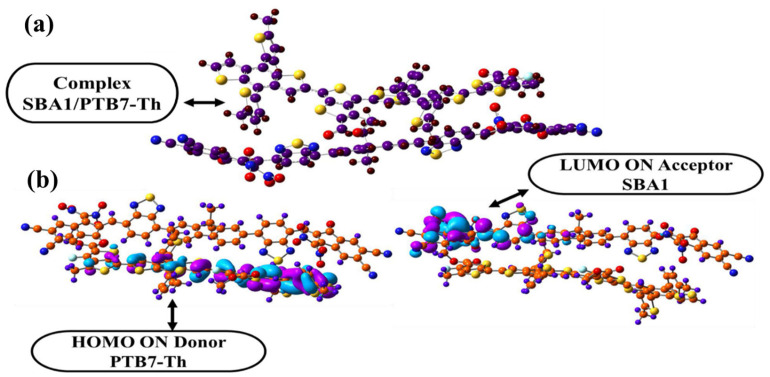
(**a**) The complex between NFA SBA1 and polymer donor PTB7-Th, and (**b**) frontiers molecular orbitals of donor:acceptor complex representing the charge transfer process.

To assess the enhanced optoelectronic capabilities of the designed molecules (SBA1–SBA8), a charge transfer analysis was carried out. The results validate PTB7-Th’s application as a donor polymer. The lowest values of *V*_oc_ and E_g_ are found in the SBA1 molecule. It is therefore SBA1 is used to study charge transfer in the complex (SBA1–PTB7-Th) forms. For charge transfer analysis, the complex of the SBA1 molecule is formed with renowned donor polymeric material PTB7-Th. The interaction between the SBA1 acceptor and the PTB7-Th donor takes place in such a pattern that the overall orientation of PTB7-Th becomes parallel to the SBA1 molecule in which the functional group side of PTB7-Th orients toward the acceptor part of SBA1, as in Figure 16a.

The electronic structure of the SBA1:PTB7-Th complex is significantly affected by the relative orientation of SBA1 and PTB7-Th, which facilitates the transfer of charge among donor and acceptor segments; their FMOs are shown in Figure 16b. The FMO investigation, which is concentrated on the SBA1:PTB7-Th complex at the B3LYP/6–31G (d, p) level, is illustrated in Figure 16b.

### 2.17. Natural Bonding Orbital Analysis

According to the NBO analysis, PTB7-Th (donor polymer) and SBA1 molecules are charge transfer mediums. The C-C bond of PTB7-Th donates electron density to the C-H bond of SBA1, which results in a charge shift. In addition, the lone pair of oxygen and sulfur atoms in PTB7-Th also plays a similar role in transferring charge to the C-H and C-N bonds of SBA1. The energy required for these charge transfer processes is relatively low (0.11–1.40), suggesting it is a facile process. The proximity of carbon, hydrogen, and sulfur atoms in these molecules facilitates an efficient interfacial charge transfer mechanism.

Table 10 shows the atoms involved in the charge-shifting mechanism. The E2 shows the energy needed for this charge-shifting mechanism at the donor-acceptor molecule contact. This research clarifies how the transfer of electron density between specific bonding and antibonding orbitals of PTB7-Th and SBA1 molecules interacts. For example, C_24_–H_27_ of the SBA1 acceptor receives electron density from the LP (S_(108)_) of PTB7-Th (donor) using 0.61 kcal/mol of energy. The lone pair (S_114_) of the PTB7-Th (donor polymer) uses 0.70 kcal/mol energy to transfer electron density to the C_1_–H_7_ bond of the SBA1. Similar outcomes are seen when 1.88 kcal/mol of energy is used by the donor polymer’s LP (O_197_) to transfer electron density to the S_39_–N_41_ of SBA1.

To comprehend the mechanism of interaction between PTB7-Th and SBA1, it should be emphasized that identifying the particular donor and acceptor components involved in this charge transfer process is crucial. Researchers with a deeper comprehension of this process can achieve improved design and optimization of organic photovoltaic materials.

## 3. Materials and Methods

### Computational Details

The Gaussian 09 W program was used to perform quantum calculations [41]. Gauss View 5.0 program was used to make all input files [42]. DFT calculations at six different functionals, B3LYP [43], ωB97XD [44], CAM-B3LYP [45], M06-2X [46], and MPW1PW91 [47] with the conjunction of 6–31G (d, p) basis set, was performed for optimization of reference molecule R [14]. The absorption maximum (λ_max_) values were estimated from TD-DFT computations at the above mentioned six functions. To choose the most applicable functional, we compared the λ_max_ value of the R computed theoretically with the experimentally determined λ_max_ value reported as shown in Figure 2. Findings from B3LYP/6–31G (d, p) exhibited the closest match with the experimentally published values, providing the basis for further computations of designed molecules utilizing this functional and basis set combination. The UV-Vis spectral characteristics of the reference compound R and the designed molecules SBA1–SBA8 were assessed using the CPCM model with chloroform as the solvent, employing the TD-DFT/B3LYP/6–31G (d, p) computational approach. Additionally, we conducted an extensive analysis of Frontier Molecular Orbitals (FMOs), density of states (DOS), reorganization energies (RE), transition density matrices (TDM), and open-circuit voltage (*V*_oc_) for both R and SBA1–SBA8 at the DFT/B3LYP/6–31G (d, p) level of theory. Reorganization energies, in particular, are crucial as described by Marcus’ theory, which categorizes them into two types: internal (λ_int_) and external (λ_ext_) reorganization energies. The internal reorganization energy is associated with the structural changes within a molecule, while the external reorganization energy pertains to the influence of the surrounding environment. In this study, the focus was on the internal reorganization energy, with the external reorganization energy being considered negligible. The following equations are used for the reorganization energies of λ_e_ (electron) and λ_h_ (hole) [48].

(13) $$\lambda e=\left[E_{0}^{-}-E_{-}\right]+\left[E_{0}^{-}-E_{0}\right]$$

(14) $$\lambda h=[E_{0}^{+}-E^{+}]+[E_{0}^{+}-E_{0}]$$

In this equation, E_0_ indicates single point energy values of anion and cations, respectively, obtained through simple optimization and neutral molecules. The $E_{0}^{+}\;$ and $\;E_{0}^{-}$ represent cationic and anionic energies via optimized geometries of cations and anions. $E_{+}^{0}\;$ and $E_{-}^{0}$ represent neutral molecule energies through anion and cation optimization. The energy of a neutral molecule in its ground state is denoted by the symbol E_0_. All results obtained from Gaussian Calculations were executed using Origin8.0 [49], VMD, PyMolyze 1.1 [50], Multiwfn program [51], chem craft [52], and Avogadro programs [22,53].

## 4. Conclusions

In conclusion, we investigated a series of eight small-molecule-based rhodanine-flanked NFAs for OSCs. These materials possess deeper HOMOs and narrower bandgaps as compared to the synthetic R molecule. Moreover, the studied molecules (SBA1–SBA8) presented an improved light absorption phenomenon, and their optical characteristics are in the range of 643.14 to 722.43 nm compared to the R molecule’s 588.30 nm, respectively. The optical characteristics have been carried out both in the gaseous and solvent phase medium and the choice of solvent (O-xylene) is made based on the solvent used for the experimental study of the R molecule. The light harvesting efficiency of these proposed materials (SBA1–SBA8) has also been increased due to the various structural modifications on both sides of the R molecule, ensuring our efficient molecular modeling strategy. Also, the calculated dipole moments show higher values in the excited state compared with the ground state analysis, suggesting less charge recombination and improved solubility in O-xylene solvent. The photovoltaic analysis revealed that these materials could produce improved *V*_oc_ and comparable FF values when integrated into photovoltaic devices, and thus, these materials have the potential to further improve the PCE of the devices. To determine the most promising candidate for BHJ-OSCs, we further analyzed the electronic and charge transfer characteristics in combination with PTB7-Th as the donor material. Among the studied molecules, SBA5 and SBA7 exhibit the most favorable energy level alignment, enhanced electron mobility, and optimal frontier molecular orbital distribution for efficient charge separation. Additionally, their higher exciton dissociation rates and better compatibility with PTB7-Th suggest their strong potential as acceptor materials in BHJ-OSCs. Therefore, we recommend SBA5 and SBA7 as the most promising candidates for synthesis and experimental validation and suggest that they be employed for OSCs, particularly when paired with polymer donor PTB7-Th as the donor material. These findings provide a valuable framework for the rational design of next-generation NFAs to enhance OSCs performance.

## Figures and Tables

**Table 1:** Theoretically calculated E_g_, energy of HOMO and LUMO of SBA1–SBA8, and synthetic R molecule.

Molecules	HOMO (E_HOMO_) (eV)	LUMO (E_LUMO_) (eV)	E_g_ = E_LUMO_ − E_HOMO_ (eV)
R	−5.73	−3.24	2.49
SBA-1	−6.38	−4.26	2.11
SBA-2	−6.02	−3.68	2.34
SBA-3	−6.22	−4.03	2.19
SBA-4	−6.36	−4.18	2.18
SBA-5	−6.06	−3.76	2.30
SBA-6	−6.16	−3.87	2.29
SBA-7	−6.26	−4.01	2.26

**Table 2:** Significant percentages of donors and acceptors contribute to generating the HOMO and LUMO of the standard R and the SBA1–SBA8 series.

Molecules	Orbitals	Donor (%)	Acceptor (%)
R	HOMO	8.7	91.3
	LUMO	46	54
SBA1	HOMO	5.2	94.8
	LUMO	57.9	42.1
SBA2	HOMO	6.2	93.8
	LUMO	56.7	43.3
SBA3	HOMO	5.9	94.1
	LUMO	53.4	46.6
SBA4	HOMO	6.0	94.0
	LUMO	55.8	44.2
SBA5	HOMO	7.1	92.9
	LUMO	54.2	45.8
SBA6	HOMO	7.2	92.8
	LUMO	56	44.0
SBA7	HOMO	6.3	93.7
	LUMO	55.3	44.7
SBA8	HOMO	5.3	94.7
	LUMO	55.8	44.2

**Table 3:** Theoretically calculated dihedral angles of SBA1–SBA8 and synthetic R molecule.

Molecules	$\phi_{1}$ (C24C25C3C2)	$\phi_{2}$ (C11C12C32C33)
R	−34.98°	33.48°
SBA-1	35.24°	−32.11°
SBA-2	34.80°	−32.64°
SBA-3	−32.68°	−34.18°
SBA-4	−33.85°	32.03°
SBA-5	34.36°	−32.50°
SBA-6	−32.49°	34.28°
SBA-7	−32.09	34.57
SBA-8	33.86	−32.14

**Table 4:** Calculated UV-visible absorption, excitation energy, and oscillation strength of SBA1–SBA8, and synthetic R molecule in chloroform solvent.

Molecules	DFT Calculated λ_max_ (nm)	Experimental λ_max_ (nm)	E_x_ (eV)	*f*_os_	Major MO Assignment
R	588.30	580	2.11	1.83	HOMO > LUMO (97%)
SBA-1	719.88		1.72	1.70	HOMO > LUMO (94%)
SBA-2	643.14		1.93	1.58	HOMO > LUMO (93%)
SBA-3	693.93		1.79	2.02	HOMO > LUMO (94%)
SBA-4	694.12		1.79	1.91	HOMO > LUMO (96%)
SBA-5	654.75		1.89	2.08	HOMO > LUMO (97%)
SBA-6	653.44		1.90	2.06	HOMO > LUMO (97%)
SBA-7	663.69		1.87	1.70	HOMO > LUMO (94%)
SBA-8	722.43		1.72	1.85	HOMO > LUMO (96%)

**Table 5:** The calculated energy gap, first singlet excitation energy, and binding energy of SBA1–SBA8 and the synthetic R molecule.

Molecules	E_(H-L)_ (eV)	E_opt_ (eV)	E_b_ = E_g_ − E_opt_ (eV)
R	2.49	2.18	0.31
SBA-1	2.11	1.85	0.26
SBA-2	2.34	2.03	0.31
SBA-3	2.19	1.89	0.30
SBA-4	2.18	1.90	0.28
SBA-5	2.30	1.99	0.31
SBA-6	2.29	1.98	0.31
SBA-7	2.26	1.97	0.29
SBA-8	2.13	1.84	0.29

**Table 6:** Chemical potential softness, hardness, nucleophilicity, and total charge transfer of SBA1–SBA8 and synthetic R molecule.

Molecules	μ (eV)	η (eV)	S (eV)	χ (eV)	ω (eV)	ΔNmax (e)
R	−4.49	1.25	0.40	4.49	8.08	3.60
SBA-1	−5.32	1.06	0.47	5.32	13.38	5.03
SBA-2	−4.85	1.17	0.43	4.85	10.06	4.15
SBA-3	−5.13	1.10	0.46	5.13	11.98	4.68
SBA-4	−5.27	1.09	0.46	5.27	12.72	4.83
SBA-5	−4.91	1.15	0.43	4.91	10.47	4.27
SBA-6	−5.01	1.15	0.44	5.01	10.97	4.38
SBA-7	−5.13	1.13	0.44	5.13	11.68	4.55
SBA-8	−5.32	1.06	0.47	5.32	13.33	5.01

**Table 7:** Theoretically computed values of *V*_oc_, fill factor, and PCE for rhodanine flanked-based reference R and developed SBA-1–SBA7 efficient organic photovoltaics.

Molecules	*V*_oc_	FF%	PCE
R	2.26	93.84%	4.4%
SBA-1	1.24	90.07%	9.7%
SBA-2	1.82	91.32%	14.7%
SBA-3	1.47	90.57%	11.7%
SBA-4	1.32	92.41%	10.5%
SBA-5	1.74	92.00%	14.1%
SBA-6	1.63	91.42%	13.1%
SBA-7	1.49	90.07%	11.9%
SBA-8	1.24	93.84%	9.8%

**Table 8:** Computed charge transfer parameters: dipole HDI, EDI, H index (Å), D index (Å), t index (Å), integral of the hole, electron, transition density, and HCT for the SBA1–SBA8 and synthetic R molecule at the B3LYP/6–31G (d, p) level of theory.

Molecules	HDI (eV)	EDI (eV)	Integral Hole (amu)	Integral Electron (amu)	Integral TD (amu)	t Index (eV)	H Index (eV)	D Index (eV)	HCT (eV)
R	3.79	4.52	0.9696	0.9696	0.00016	−1.84	8.67	0.40	2.24
SBA-1	4.32	11.2	0.9084	0.9079	−0.00015	0.93	6.49	6.97	6.03
SBA-2	4.51	3.37	0.8309	0.8311	−0.00008	−5.93	9.11	2.72	8.66
SBA-3	5.72	4.76	0.8809	0.8813	0.00001	10.64	7.51	17.7	7.08
SBA-4	3.45	4.28	0.8113	0.8116	0.00007	0.17	7.31	6.98	6.81
SBA-5	3.53	4.03	0.8892	0.8894	−0.00009	−4.35	8.28	3.35	7.70
SBA-6	4.36	5.66	0.9260	0.9257	−0.00002	−0.63	6.72	5.66	6.29
SBA-7	7.27	3.94	0.7801	0.7797	0.00008	−1.60	6.76	4.59	6.19
SBA-8	5.87	4.00	0.9347	0.9349	0.00002	−1.86	6.96	1.43	3.29

**Table 9:** The computed values of quadrupole moment of all investigated compounds SBA1–SBA8 along with reference R.

Molecules	Q_20_ (Quadrupole) Values
R	358.8364
SBA-1	452.9155
SBA-2	494.1883
SBA-3	490.0759
SBA-4	442.6615
SBA-5	449.5120
SBA-6	451.0538
SBA-7	451.0321
SBA-8	431.4807

**Table 10:** Energy and charge transfer mechanism between donor polymer and acceptor molecule.

Donor (PTB7-Th) NBO	Acceptor (SBA1) NBO	E2 kcal/mol
BD (2) C100–C105	BD*(1) C24–H27	0.11
BD (2) C109–C110	BD*(2) C22–C23	0.21
BD (2) C196–O197	BD*(1) S39–N41	0.20
BD (1) C146–H148	BD*(2) N93–O94	0.16
LP (2) S108	BD*(1) C24–H27	0.61
LP (2) S111	BD*(1) C44–H46	1.40
LP (1) S114	BD*(1) C1–H7	0.70
LP (2) S135	BD*(2) C72–O75	0.29
LP (2) S195	BD*(2) C32–N40	0.27
LP (1) O197	BD*(1) S39–N41	1.88
LP (2) O197	BD*(1) S39–N41	1.77
LP (1) S111	BD*(1) C44–H46	0.30

## Data Availability

The data presented in this study are provided in the Supplementary Materials.

## Supplementary Materials

The following supporting information can be downloaded at: https://www.mdpi.com/article/10.3390/ijms26073314/s1.
