# Supplementary material for: Deciphering the Role of Rhodanine Flanked Non-Fullerene Acceptor Molecules for Efficient Organic Photovoltaics
Source: Int J Mol Sci. 2025 Apr 2;26(7):3314. doi: 10.3390/ijms26073314 (PMC11989678; doi:10.3390/ijms26073314)
Supplement: Supplementary file 1 [file ijms-26-03314-s001.zip › ijms-3504757-supplementary.pdf]

Supporting Information

Deciphering the Role of Rhodanine Flanked Non-Fullerene Acceptor Molecules for Efficient Organic Photovoltaics

Zobia Irshad [1†], Muzammil Hussain [2†], Riaz Hussain [2] and Muhammad Adnan [1*]

[1] *Graduate School of Energy Science and Technology, Chungnam National University, Daejeon, 34134, Republic of Korea.*

[2] *Department of Chemistry, University of Okara, 56300, Pakistan.*

[†] *Contributed equally.*

Correspondence: adnan5750@gmail.com; adnan@cnu.ac.kr

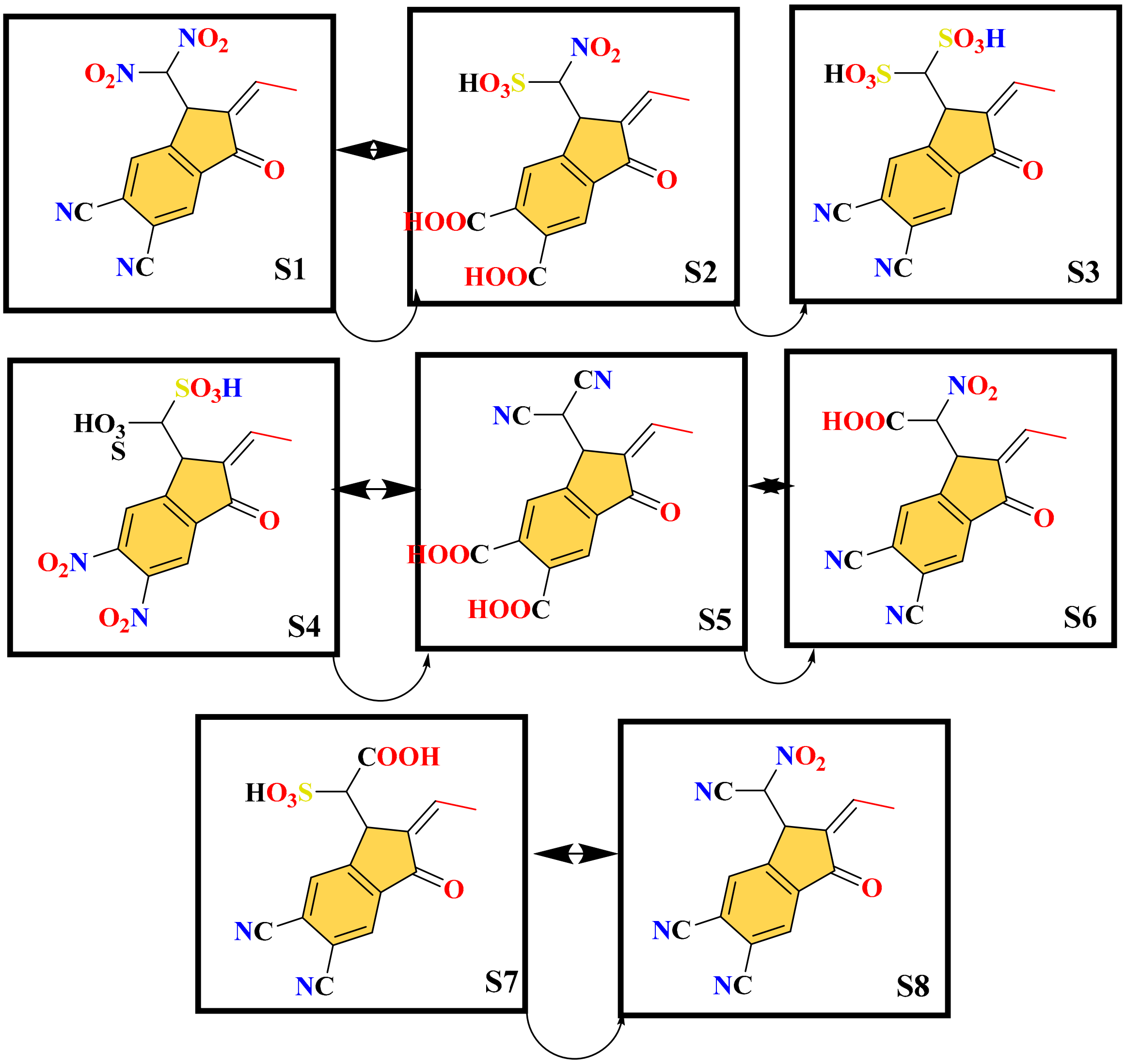

Figure S1. End-capped groups utilized in the modification of the designed structures.

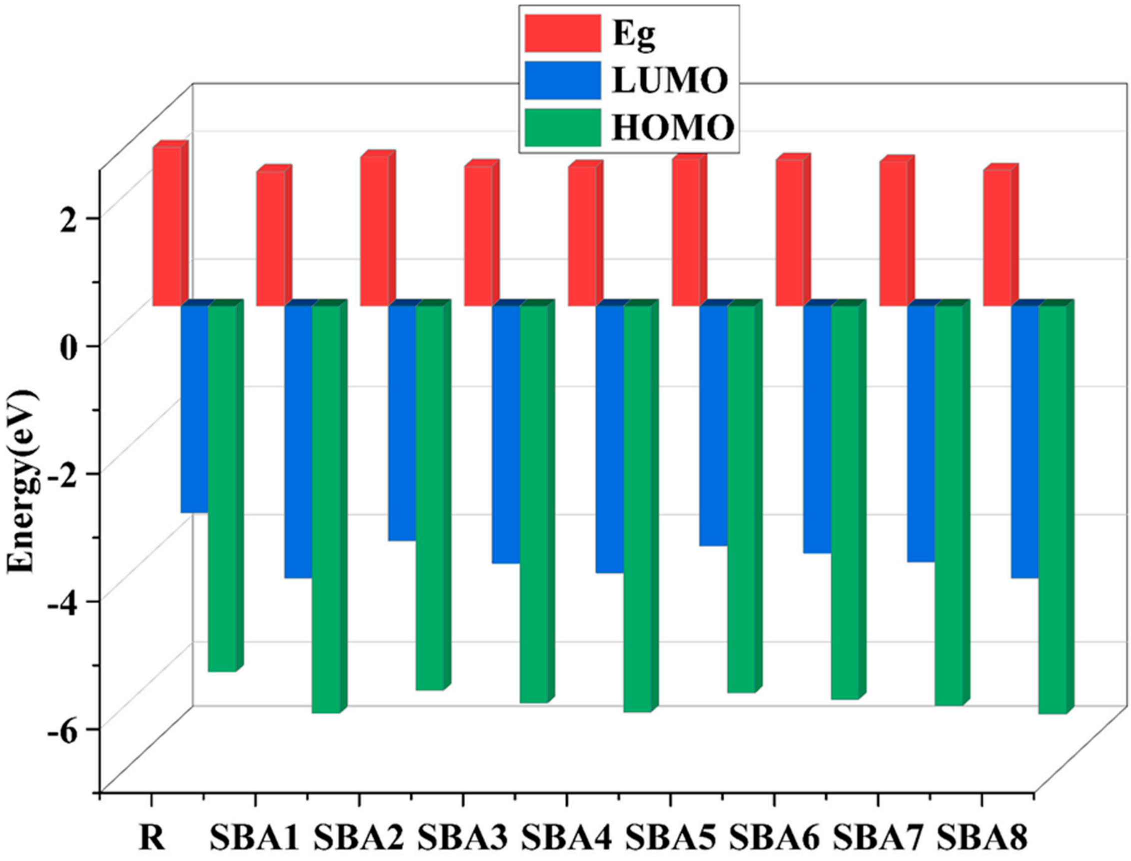

Figure S2. The energy gap between the HOMO and LUMO of the reference and the designed molecules.

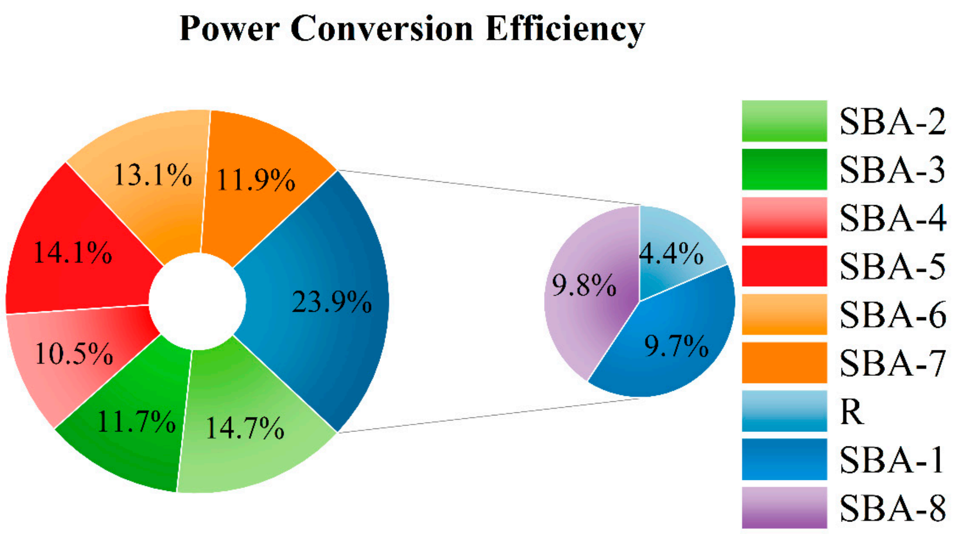

Figure S3. The estimated power conversion efficiency of all designed molecules (SBA1-SBA8) and the reference molecule.

Table S1. Calculated UV-visible absorption, excitation energy, oscillation strength of SBA1-SBA8, and synthetic R molecule in the Gas Phase.

Molecules	DFT Calculated λ_{max} (nm)	Experimental λ_{max} (nm)	E_x (eV)	f_{os}	Major MO Assignment
R	569.47	580	2.18	1.57	HOMO > LUMO (98%)
SBA-1	671.20		1.85	1.60	HOMO > LUMO (95%)
SBA-2	611.72		2.03	1.54	HOMO > LUMO (95%)
SBA-3	655.13		1.89	1.86	HOMO > LUMO (97%)
SBA-4	653.93		1.90	1.80	HOMO > LUMO (98%)
SBA-5	621.69		1.99	1.90	HOMO > LUMO (98%)
SBA-6	625.01		1.98	1.90	HOMO > LUMO (98%)
SBA-7	628.88		1.97	1.64	HOMO > LUMO (95%)
SBA-8	673.39		1.84	1.74	HOMO > LUMO (98%)

Table S2. Computed LHE in Solvent phase for each Rhodanine Flanked-based acceptor under study.

**Molecules**	**LHE Solvent**	**LHE Gas**
**R**	0.9852	0.9731
SBA-**1**	0.9800	0.9749
SBA-**2**	0.9737	0.9712
SBA-**3**	0.9905	0.9862
SBA-**4**	0.9877	0.9842
SBA-**5**	0.9917	0.9874
SBA-**6**	0.9913	0.9874
SBA-7	0.9800	0.9771
SBA-8	0.9859	0.9818

Table S3. Computed LHE in gaseous phase for each Rhodanine Flanked-based acceptor under study.

**Molecules**	f_{os}	**LHE**
**R**	1.57	0.9731
SBA-**1**	1.60	0.9749
SBA-**2**	1.54	0.9712
SBA-**3**	1.86	0.9862
SBA-**4**	1.80	0.9842
SBA-**5**	1.90	0.9874
SBA-**6**	1.90	0.9874
SBA-7	1.64	0.9771
SBA-8	1.74	0.9818
